# The association between tuberculin skin test result and active tuberculosis risk of college students in Beijing, China: a retrospective cohort study

**DOI:** 10.1186/s12879-019-4238-2

**Published:** 2019-07-12

**Authors:** Demin Cao, Zhiguo Zhang, Zhen Yang, Shubo Ma, Zhaogang Sun, Huijuan Duan, Baoli Zhu, Fei Zhao

**Affiliations:** 1 CAS Key Laboratory of Pathogenic Microbiology and Immunology, Institute of Microbiology, Chinese Academy of Sciences, Beijing, 100101 China; 2 University of Chinese Academy of Sciences, Beijing, 100049 China; 3 Beijing Changping institute for tuberculosis prevention and treatment, Beijing, 102200 China; 4 Beijing Tuberculosis and Thoracic Tumor Research Institute, Beijing, 101149 China; 5 Clinical Trials and Research Center, National Center for Geriatrics of Beijing Hospital, Beijing, 100730 China

**Keywords:** Tuberculosis risk, Tuberculin skin test, College students

## Abstract

**Background:**

About 10% latent tuberculosis infections (LTBI) would progress to active tuberculosis (TB), if left prophylactic therapy. Tuberculin skin test (TST) is the most widely used method for LTBI screening in the school of China. However, for college students, the association between TST reaction size and active TB risk was unclear.

**Methods:**

We conducted a retrospective study to assess whether the TST reaction size would predict active TB during the next two years after TST screening for college students. Multivariable Cox regression was performed to identify the size of TST reaction and other factors associated with active TB risk.

**Results:**

A total of 67292 college students in Beijing, China were included in this study; 8021 (11.92%) individuals were TST positive (≥10 mm), and 3879 (5.76%) of them were strong TST positive (≥15 mm). During the two years of follow-up, 26 active TB cases were reported in 134575 person-years with an incidence rate of 19.32 (95% CI: 12.61–28.32) per 100000 person-years. The adjusted hazard ratios (HR) (95% CI) were 1.094 (0.247~4.846), 3.644 (1.188~11.179), 6.832 (2.436~19.163) and 9.768 (2.203~43.315) of cohorts with the TST reaction size intervals 5~9, 10~14, 15~20 and ≥ 20 mm, respectively, compared to cohort with interval 0~4 mm. Besides, the adjusted HR (95% CI) was 3.593 (1.354~9.537) of males compared to females.

**Conclusions:**

This study indicated that the risk of active TB increased in college students when the TST reaction size was ≥10 mm, and males had a higher risk compared to females.

**Electronic supplementary material:**

The online version of this article (10.1186/s12879-019-4238-2) contains supplementary material, which is available to authorized users.

## Background

Worldwide, it is estimated that one-third of the world’s population is infected with *Mycobacterium tuberculosis* (*Mtb*). In 2017, about 10 million people suffered from tuberculosis, and 12–14% of them died. Wherein China has the second largest number of TB patients (best estimate, 88.9 million; range, 76.1~10.30 million) in the world [1]. About 5–10% of people with LTBI would suffer from active TB. But approximately 60–90% such activation of TB would be prevented by early diagnosis and preventing treatment [2, 3].

School is a people-intensive place, in which students have been in prolonged face to face contact with each other. It is one of the most common places that reported for the community-based outbreak of TB in China [4, 5]. It is significant to implement early diagnosis and prophylactic therapy in the prevention of school tuberculosis. At present, tuberculin skin test, based on purified protein derivative (PPD) induced delayed-type hypersensitivity, is still the main means in *Mtb* infection screening in China, because of its handleability and inexpensive price.

Several studies have been performed among primary school students to estimate the risk of TB disease according to TST reaction size [6, 7]. However, very few studies focused on the association of TST reaction size and TB risk among college students, who were during or past-puberty with great changes of physiology and behavior. It was reported that the incidence of LTBI, as well as TB, for the people beyond the age of 15 years is higher than for them under that age [6, 8]. In this study, we aim to investigate the relationship between the risk of active TB and the intensity of the TST response of college students.

## Methods

### Study design and participants

We performed a retrospective cohort study of the college freshmen TB infection investigation of 20 colleges, located in Changping district, Beijing, China. The baseline examination took place in 2013–2016. Follow-up for all individuals started at the time they received TST, mainly in mid-September each year. We ended follow-up for each individual with 2 years follow-up. The data were excluded if any of the following conditions exist: active TB diagnosis in freshmen TB screening, having active TB history or missing TST results.

### Data collection and procedures

Beijing Changping institute for tuberculosis prevention and treatment (BCITPT) is the public health institution mainly responsible for TB prevention and control in Changping district. Raw records of colleges’ freshmen TB screening of 2013–2016, which contained the age, sex, ethnicity, region, TST reaction size, and diagnosis result, were obtained from BCITPT. Data on reported TB cases of the colleges in Changping district were retrieved from national TB reporting system from 2013 to 2018. Besides, the incidence time of each TB case was collected for further analyses.

According to the medical records of BCITPT, TST was conducted with 2 units of tuberculin (TB-PPD, Beijing Sanroad biological products co., Ltd) on the left forearm using the intradermal technique for each college freshman. And results were measured and recorded after 72 h of TST. For students with the strong positive reaction of TST (induration size ≥15 mm or the presence of blister or necrosis), the subsequent medical diagnosis was performed using X-ray, *Mtb* culture of sputum and microscopy examination of sputum smears and so on. The individuals with TST reaction size ≥20 mm (886 ones in this study) were advised to receive preventive therapy. However, it was non-obligatory, a few students received preventive therapy, and because it needs three months of treatment, few ones successfully complete treatment. In this study, no one was excluded from the analysis due to receiving LTBI treatment. In the follow-up years, the student who was suspected to be TB case would be taken to BCITPT by the head teachers of classes of colleges in the first time, and all the suspected active TB cases in Changping district were confirmed and treated in BCITPT. They all accepted X-ray examination, microscopy examination of sputum smears and *Mtb* culture of sputum. The diagnosis and classification of TB were based on *the national guidelines for diagnosis for pulmonary tuberculosis (WS 288—2008)* and *Classification of tuberculosis (WS 196—2001)* [9, 10].

### Statistical analyses

All analyses were performed using R 3.5.2 in this study. Pearson’s Chi-square test or Fisher’ exact test, where appropriate, was used to compare the frequency of categorical variables between the cohorts. The incidence rate (IR) and 95% confidence interval (CI) for each cohort were calculated. A 2-tailed *P* value of less than 0.05 was used to identify the statistical significance for all tests. Separately, cumulative incidence curves for different categories of sex and TST reaction size levels were plotted by using the Kaplan-Meier method, and the log-rank test was used to compare the curves. Subsequently, to identify the potential factors related to TB incidence, regression analysis based on Cox’s proportional hazards model was applied to estimate the unadjusted and adjusted hazard ratio (HR) and 95% CI. The univariates with *P* values less than 0.10 were then candidates included in multivariate Cox’s regression analysis. Besides, HR analyses of subgroups of males and females were performed with the same methods. The absolute risk of active TB (1/100000) of different TST reaction sizes was calculated and plotted.

## Results

As shown in Table 1, a total of 68288 individuals were included in the enrollment TB screening project of college students during 2013–2016, and 67428 of them actually participated. There were no significant differences in age, sex, and region between those who agreed to participate and those who didn’t. After excluding 88 individuals diagnosed as active TB and 48 ones who had active TB history, 67292 (98.54%) individuals were included in the final follow-up cohort. They came from 31 provincial divisions of China, including Beijing (16234, 24.12%), Hebei (3922, 5.83%), Shandong (3125, 4.64%), He’nan (2889, 4.30%), Xinjiang (2086, 3.10%) and so on. There were 36351 (54.02%) males and 30941 (45.98%) females. And the mean (standard deviation, SD) age of the students was 19.52 (1.26) years at baseline (Additional file 1: Table S1).

**Table 1:** Baseline characteristics of students

Year	Enrollment of freshmen	# of follow-up	Sex		Age (means ± SD)	TST positive(%)	Strong TST positive(%)
			Male(%)	Female(%)			
2013	14899	14592	45.59	54.41	19.78 ± 1.53	10.85	5.10
2014	20081	19823	51.70	48.30	19.52 ± 1.13	10.60	4.61
2015	18914	18659	58.63	41.37	19.37 ± 0.95	13.53	6.92
2016	14394	14218	59.86	40.14	19.35 ± 1.04	12.74	6.54
Total	68288	67292	54.02	45.98	19.52 ± 1.26	11.92	5.76

TST positive: induration size ≥10 mm or the presence of blister or necrosis

Strong TST positive: induration size ≥15 mm or the presence of blister or necrosis

There were 8021 (11.92%) TST positive individuals (induration size ≥10 mm or the presence of blister or necrosis), and about half of them (3879, 5.76%) were strong TST positive (induration size ≥15 mm or presence of blister or necrosis) (Table 1). For TST negative individuals, reaction sizes of them were mainly distributed in 0~1 (51401, 76.39%), 4~5 (5278, 7.84%) and 8~9 (1328, 1.97%); for TST positive individuals, the reaction sizes of them were mainly distributed in 10~11 (2849, 4.23%), 12~13 (1279, 1.90%) and 14~15 (1925, 2.86%) (Additional file 1: Table S2). There were significant differences between the students with TST positive and TST negative in sex, ethnicity, and region (Additional file 1: Table S3). Specifically, males had a higher percentage of TST positive compared to females (12.18% vs. 11.61%); non-Han cohort had a higher percentage of TST positive compared to Han cohort (15.86% vs. 11.45%); the distribution trends of regions of TST positive was high in the west and low in the east (west: 14.22%, middle: 12.39%, east: 10.55%).

There were 26 active TB cases during the 2 years of follow-up. Three of them were diagnosed by microscopy examination of sputum smears; three of them were diagnosed by *Mtb* culture of sputum, and the others were diagnosed due to TB-related symptoms. During the one-year follow-up period, 9 cases of active TB were recorded in 67292 person-years with an incidence rate of 13.37 (95% CI: 6.11~25.40) per 100000 person-years. And for two-year follow up, 26 active TB cases were recorded in 134575 person-years with an incidence rate of 19.32 (95% CI: 12.61–28.32) per 100000 person-years. However, no significant difference in incidence rate was observed between the one-year and two-year follow up. Compared with the incidence rate of female (8.08 per 100000 person-years, 95% CI: 2.62~18.86), male individuals (28.89 per 100000 person-years, 95% CI: 17.88~44.17) had a higher incidence rate (*P* = 0.01). The comparison among subgroups who came from different regions of China indicated that those from western China had the highest incidence rate, which might be due to the higher rate of LTBI in those regions at baseline. But no significant difference in the incidence rate was observed among students from different regions of China, as well as Han and non-Han ethnicity (Table 2). According to the Chi-squared test for trend in proportions, it was observed that the incidence rate showed an increasing trend with increasing of TST reaction size (chi-squared = 24.037, *P* < 0.001).

**Table 2:** Factors associated with active TB incidence in follow-up individuals

	# of individuals	TB events/follow-up person-years	IR^a^(95% CI)	*P* for chi-square test	Univariate Cox regression		Multivariate Cox regression	
					HR (95% CI)	*P* value	Adjusted HR(95% CI)	*P* value
Sex				0.01				
Female	30941	5/61881	8.08 (2.62~18.86)		Reference		Reference	
Male	36351	21/72694	28.89 (17.88~44.17)		3.576 (1.348~9.483)	0.01	3.593 (1.354~9.537)	0.01
Ethnicity				0.86				
Han	60086	23/120164	19.14 (12.12~28.73)		Reference		Reference	
Others	7206	3/14411	20.82 (4.29~60.85)		1.088 (0.3266~3.622)	0.89		
Region				0.21				
East	33692	14/67377	20.78 (11.35~34.87)		Reference		Reference	
Middle	17069	3/34138	8.79 (1.81~25.69)		0.423 (0.121~1.472)	0.17		
West	16531	9/33060	27.22 (12.44~51.70)		1.310 (0.567~3.027)	0.52		
TST response (induration size, mm)				< 0.001				
0~4	51963	13/103923	12.51 (6.65~21.40)		Reference		Reference	
5~9	7308	2/14615	13.68 (1.66~49.44)		1.094 (0.247~4.848)	0.91	1.094 (0.247~4.846)	0.91
10~14	4217	4/8431	47.44 (12.90~121.50)		3.794 (1.237~11.635)	0.02	3.644 (1.188~11.179)	0.02
15~19	2918	5/5834	85.70 (27.77~200.03)		6.853 (2.443~19.223)	< 0.001	6.832 (2.436~19.163)	< 0.001
≥ 20	886	2/1772	112.87 (13.66~407.79)		9.032 (2.038~40.025)	0.004	9.768 (2.203~43.315)	0.003

*IR* Incidence rate, *HR* Hazard ratio, *CI* Confidence interval

^a^ Per 100 000 person years

**Fig. 1:**
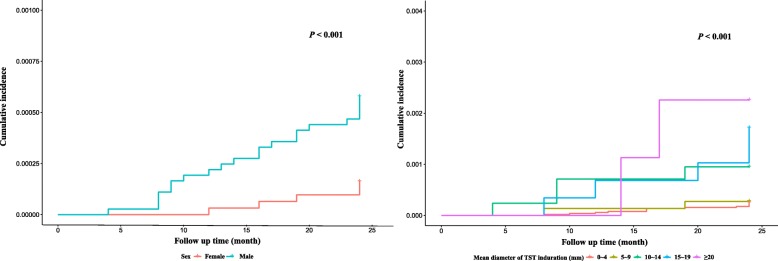
Cumulative incidence curves of active TB estimated by Kaplan-Meier method. (**a**) Cumulative incidence curve analysis of different intervals of the mean diameter of TST induration (0~4, 5~9, 10~14, 15~19 and ≥ 20) (**b**) Cumulative incidence curve analysis of sex

**Fig. 2:**
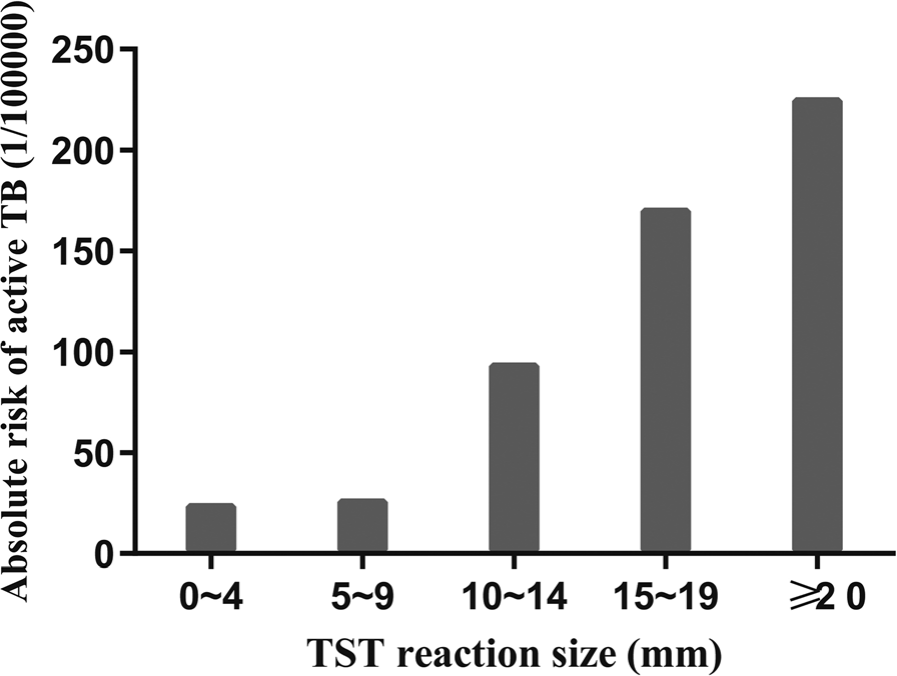
The absolute risk of active TB of different TST reaction sizes

Factors that significantly related to active TB incidence were sex and TST response. As shown in Fig. 1, the cumulative incidence of active TB had a significant difference between males and females (Fig. 1a), as well as subgroups with different TST reaction size levels (Fig. 1b). The significant variables for the active TB were summarized in Table 2. After adjusted with other factors, the males were found to have a higher risk of TB development by comparing with the females, with an adjusted HR 3.359 (95% CI: 1.354~9.537). For the TST response intensity, there was no significant difference of active TB risk between individuals with mean diameter of TST induration 0~4 mm and 5~9 mm (adjusted HR: 1.094, [95% CI, 0.247~4.846], *P* = 0.91). However, when the induration size was greater than 10 mm (10~14, 15~19 and ≥ 20, respectively), the adjusted HR (95% CI) showed an upward trend (3.644 [1.188~11.179], 6.832 [2.436~19.163] and 9.768 [2.203~43.315], respectively) (Table 2). From 0~4 to ≥20, the absolute risk of active TB (1/100000) were 25.02, 27.38, 94.85, 171.35, and 225.73, respectively. As can be seen from Fig. 2, there was little difference in the absolute risk of active TB between TST reaction size 0~4 mm and 5~9 mm, but it was increased rapidly with TST reaction size greater than 10 mm. Moreover, subgroups analyses of males and females showed that HR of the cohort with TST reaction size ≥10 mm was 5.41 (95% CI: 2.28~12.84, *P* < 0.001) compared to those with reaction size < 10 mm for males, and 5.08 (95% CI: 0.85~30.37, *P* > 0.05) for females. This suggested that in males, the risk of active TB was higher in college students with the TST positive results, but the difference was not significant for females.

## Discussion

In this retrospective study, we found that TST reaction size ≥10 mm in college freshmen predicted an increased risk of active TB in the subsequent 2 years of TST, compared with those with a reaction size <10 mm. Specifically, the active TB risk increased about 3 times per 5 mm increasing of the interval of TST reaction size compared to reaction size ≤5 mm, up to 9.768 times with reaction size ≥20 mm. And the distribution of the absolute risk of active TB of different induration sizes had similar features. Some previous studies also indicated that a person with a TST reaction size ≥15 mm had a high risk of active TB [6, 11]. However, college students, who were experiencing or going through puberty with great changes of physiology and behavior, were not included. Our study provided some evidence that strong TST reaction in the age of 17–21 years old could be used to predict the subsequent development of TB in two years after TST.

As the most effective and economical means for prevention from *Mtb* infection, vaccine coverage rate for bacillus Calmette-Gue’rin (BCG) has been around 97% since 1999 in China [12]. However, prior BCG vaccination had a strong influence on TST results on account of cross-reactivity among young people [13], which may lead to false positive results. Even though some more specificity and sensitivity methods have been developed, such as QuantiFERON-TB Gold, T-SPOT.TB and so forth [8, 14, 15], they have not yet been widely accepted in less developed areas because of their higher costs and complexity. Prophylactic therapy, with efficacy ranging from 60 to 90%, was necessary for LTBI persons [2, 16, 17], but not all TST positive ones. It was operable that the college students of TST reaction size ≥10 mm, about 11.92% (8021/67292) of the whole, were regarded as a high-risk population and did further screening with QFT or T-SPOT.

Furthermore, the males of students predicted a higher risk of active TB compared to females, which was consistent with the results of previous studies [18–20]. Adolescence is a bifurcation point between males and females for the incidence of some infectious disease [21]. There are two reasons that may lead to the difference in active TB incidence. One is the physiological difference between them. Sex hormones play a significant role in immune responses; the female sex hormone estrogen tends to be immune-enhancing, while the male sex hormone testosterone is usually immune-suppressive [21, 22]. The other is the behavioral difference in male and female. *Mtb* is a person-to-person transmitted pathogen mainly according to the droplet, but behavior-related exposure is usually higher among males, especially after puberty [23]. Thus, the sex-bias of immune status and *Mtb* exposure may lead to the different risk levels of incidence of active TB.

There were a few limitations in this study. At first, the absence information of some other influencing factors, such as BMI, smoking and alcohol drinking history, close contact history, comorbidity and so forth, in the raw records might lead to ignoring some risk factors and influencing the accuracy of results. Secondly, because of the missing information of students received LTBI treatment, the risk of active TB for those with TST reaction size ≥20 mm might be underestimated in this study. Thirdly. follow up time was not long enough. It was reported that TB risk decreased rapidly with time after *Mtb* infection [15, 24]. Longtime follow-up might provide more information on the relationship of TST reaction intensity and active TB risk of this population. In addition, this study was only performed in Beijing. Therefore, additional research should be done in a wider area of China with longer follow-up time to estimate the association between TST result and active TB incidence in the youth population.

## Conclusions

In summary, the intensity of TST reaction could reflect the active TB risk to some extent for college students. And males had a higher risk compared to females. This may allow us to do a better job in the prevention of pulmonary tuberculosis of college students in the future.

## Additional file

Additional file 1: **Table S1.** The age distribution of the follow-up cohort. **Table S2.** The TST reaction size distribution of follow-up cohort. **Table S3.** The characteristics of individuals with TST positive and TST negative. (DOCX 21 kb)

## Data Availability

The datasets used and/or analyzed during the current study are available from the corresponding author on reasonable request.

## Abbreviations

BCG: Bacillus Calmette-Gue’rin
BCITPT: Beijing Changping institute for tuberculosis prevention and treatment
CI: Confidence interval
HR: Hazard ratio
IR: Incidence rate
LTBI: Latent tuberculosis infections
*Mtb*: *Mycobacterium tuberculosis*
PPD: Purified protein derivative
PR: Prevalence rate
SD: Standard deviation
TB: Tuberculosis
TST: Tuberculin skin test
