# Supplementary material for: The association between tuberculin skin test result and active tuberculosis risk of college students in Beijing, China: a retrospective cohort study
Source: BMC Infect Dis. 2019 Jul 12;19:619. doi: 10.1186/s12879-019-4238-2 (PMC6626405; doi:10.1186/s12879-019-4238-2)
Supplement: Supplementary file 1 — Table S1. The age distribution of the follow-up cohort. Table S2. The TST reaction size distribution of follow-up cohort. Table S3. The characteristics of individuals with TST positive and TST negative. (DOCX 21 kb) [file 12879_2019_4238_MOESM1_ESM.docx]

Additional file 1

**Table S1 The age distribution of the follow-up cohort**

| **Age** | **Number** | **%** | **TB events** |
| --- | --- | --- | --- |
| 15 | 36 | 0.05 | 0 |
| 16 | 361 | 0.54 | 0 |
| 17 | 478 | 0.71 | 0 |
| 18 | 10978 | 16.31 | 3 |
| 19 | 24947 | 37.07 | 7 |
| 20 | 17749 | 26.38 | 9 |
| 21 | 10833 | 16.10 | 5 |
| 22 | 584 | 0.87 | 0 |
| 23 | 421 | 0.63 | 1 |
| 24 | 401 | 0.60 | 0 |
| 25 | 269 | 0.40 | 0 |
| 26 | 116 | 0.17 | 0 |
| 27 | 57 | 0.08 | 1 |
| 28 | 25 | 0.04 | 0 |
| 29 | 16 | 0.02 | 0 |
| 30 | 8 | 0.01 | 0 |
| 31 | 6 | 0.01 | 0 |
| 32 | 3 | 0.00 | 0 |
| 33 | 1 | 0.00 | 0 |
| 34 | 3 | 0.00 | 0 |
| Total | 67292 | 100.00 | 26 |

**Table S2 The TST reaction size distribution of the follow-up cohort**

| **TST result induration** | **Number** | **%** |
| --- | --- | --- |
| 0~1 | 51401 | 76.39 |
| 2~3 | 492 | 0.73 |
| 4~5 | 5278 | 7.84 |
| 6~7 | 772 | 1.15 |
| 8~9 | 1328 | 1.97 |
| 10~11 | 2849 | 4.23 |
| 12~13 | 1279 | 1.90 |
| 14~15 | 1925 | 2.86 |
| 16~17 | 635 | 0.94 |
| 18~19 | 447 | 0.66 |
| 20~21 | 585 | 0.87 |
| 22~23 | 79 | 0.12 |
| 24~25 | 114 | 0.17 |
| 26~27 | 12 | 0.02 |
| 28~29 | 11 | 0.02 |
| ≥30 | 85 | 0.13 |
| Total | 67292 | 100.00 |

**Table S3 The characteristics of individuals with TST positive and TST negative**

|  |  | **# of individuals** | **# of TST negative** | **# of TST positive** | ***P* for chi-square test** |
| --- | --- | --- | --- | --- | --- |
| **Sex** |  |  |  |  | 0.02 |
|  | Female | 30941 | 27348(88.39) | 3593(11.61) |  |
|  | Male | 36351 | 31923(87.82) | 4428(12.18) |  |
| **Ethnicity** |  |  |  |  | <0.001 |
|  | Han | 60086 | 53208(88.55) | 6878(11.45) |  |
|  | Others | 7206 | 6063(84.14) | 1143(15.86) |  |
| **Region** |  |  |  |  | <0.001 |
|  | East | 33692 | 30136(89.45) | 3556(10.55) |  |
|  | Middle | 17069 | 14955(87.61) | 2114(12.39) |  |
|  | West | 16531 | 14180(85.78) | 2351(14.22) |  |

Values are presented as number (percentage)
